# Inferred Allelic Variants of Immunoglobulin Receptor Genes: A System for Their Evaluation, Documentation, and Naming

**DOI:** 10.3389/fimmu.2019.00435

**Published:** 2019-03-18

**Authors:** Mats Ohlin, Cathrine Scheepers, Martin Corcoran, William D. Lees, Christian E. Busse, Davide Bagnara, Linnea Thörnqvist, Jean-Philippe Bürckert, Katherine J. L. Jackson, Duncan Ralph, Chaim A. Schramm, Nishanth Marthandan, Felix Breden, Jamie Scott, Frederick A. Matsen IV, Victor Greiff, Gur Yaari, Steven H. Kleinstein, Scott Christley, Jacob S. Sherkow, Sofia Kossida, Marie-Paule Lefranc, Menno C. van Zelm, Corey T. Watson, Andrew M. Collins

**Affiliations:** ^1^Department of Immunotechnology, Lund University, Lund, Sweden; ^2^Center for HIV and STIs, National Institute for Communicable Diseases, Johannesburg, South Africa; ^3^Faculty of Health Sciences, School of Pathology, University of the Witwatersrand, Johannesburg, South Africa; ^4^Department of Microbiology, Tumor and Cell Biology, Karolinska Institute, Stockholm, Sweden; ^5^Institute of Structural and Molecular Biology, Birkbeck College, University of London, London, United Kingdom; ^6^Division of B Cell Immunology, German Cancer Research Center, Heidelberg, Germany; ^7^Department of Experimental Medicine, University of Genoa, Genoa, Italy; ^8^BISC Global Inc., Boston, MA, United States; ^9^Immunology Division, The Garvan Institute of Medical Research, Darlinghurst, NSW, Australia; ^10^Fred Hutchinson Cancer Research Center, Seattle, WA, United States; ^11^Vaccine Research Center, National Institutes of Health, Washington, DC, United States; ^12^Department of Molecular Biology and Biochemistry, Simon Fraser University, Burnaby, BC, Canada; ^13^Department of Biological Sciences, Simon Fraser University, Burnaby, BC, Canada; ^14^Department of Molecular Biology and Biochemistry, Faculty of Health Sciences, Simon Fraser University, Burnaby, BC, Canada; ^15^Department of Immunology, Institute of Clinical Medicine, University of Oslo, Oslo, Norway; ^16^Faculty of Engineering, Bar Ilan University, Ramat Gan, Israel; ^17^Department of Pathology, Yale University, New Haven, CT, United States; ^18^Department of Clinical Sciences, University of Texas Southwestern Medical Center, Dallas, TX, United States; ^19^Innovation Center for Law and Technology, New York Law School, New York, NY, United States; ^20^IMGT^®^, The International ImMunoGenetics information system^®^ (IMGT), Laboratoire d'ImmunoGénétique Moléculaire (LIGM), CNRS, Institut de Génétique Humaine, Université de Montpellier, Montpellier, France; ^21^Department of Immunology and Pathology, Central Clinical School, The Alfred Hospital, Monash University, Melbourne, VIC, Australia; ^22^Department of Biochemistry and Molecular Genetics, University of Louisville, Louisville, KY, United States; ^23^School of Biotechnology and Biomolecular Sciences, University of New South Wales, Sydney, NSW, Australia

**Keywords:** immunoglobulin, allelic variation, inference, AIRR-seq, IGHV, V(D)J rearrangement

## Abstract

Immunoglobulins or antibodies are the main effector molecules of the B-cell lineage and are encoded by hundreds of variable (V), diversity (D), and joining (J) germline genes, which recombine to generate enormous IG diversity. Recently, high-throughput adaptive immune receptor repertoire sequencing (AIRR-seq) of recombined V-(D)-J genes has offered unprecedented insights into the dynamics of IG repertoires in health and disease. Faithful biological interpretation of AIRR-seq studies depends upon the annotation of raw AIRR-seq data, using reference germline gene databases to identify the germline genes within each rearrangement. Existing reference databases are incomplete, as shown by recent AIRR-seq studies that have inferred the existence of many previously unreported polymorphisms. Completing the documentation of genetic variation in germline gene databases is therefore of crucial importance. Lymphocyte receptor genes and alleles are currently assigned by the Immunoglobulins, T cell Receptors and Major Histocompatibility Nomenclature Subcommittee of the International Union of Immunological Societies (IUIS) and managed in IMGT^®^, the international ImMunoGeneTics information system^®^ (IMGT). In 2017, the IMGT Group reached agreement with a group of AIRR-seq researchers on the principles of a streamlined process for identifying and naming inferred allelic sequences, for their incorporation into IMGT^®^. These researchers represented the AIRR Community, a network of over 300 researchers whose objective is to promote all aspects of immunoglobulin and T-cell receptor repertoire studies, including the standardization of experimental and computational aspects of AIRR-seq data generation and analysis. The Inferred Allele Review Committee (IARC) was established by the AIRR Community to devise policies, criteria, and procedures to perform this function. Formalized evaluations of novel inferred sequences have now begun and submissions are invited via a new dedicated portal (https://ogrdb.airr-community.org). Here, we summarize recommendations developed by the IARC—focusing, to begin with, on human IGHV genes—with the goal of facilitating the acceptance of inferred allelic variants of germline IGHV genes. We believe that this initiative will improve the quality of AIRR-seq studies by facilitating the description of human IG germline gene variation, and that in time, it will expand to the documentation of TR and IG genes in many vertebrate species.

## Introduction

**Figure 1:**
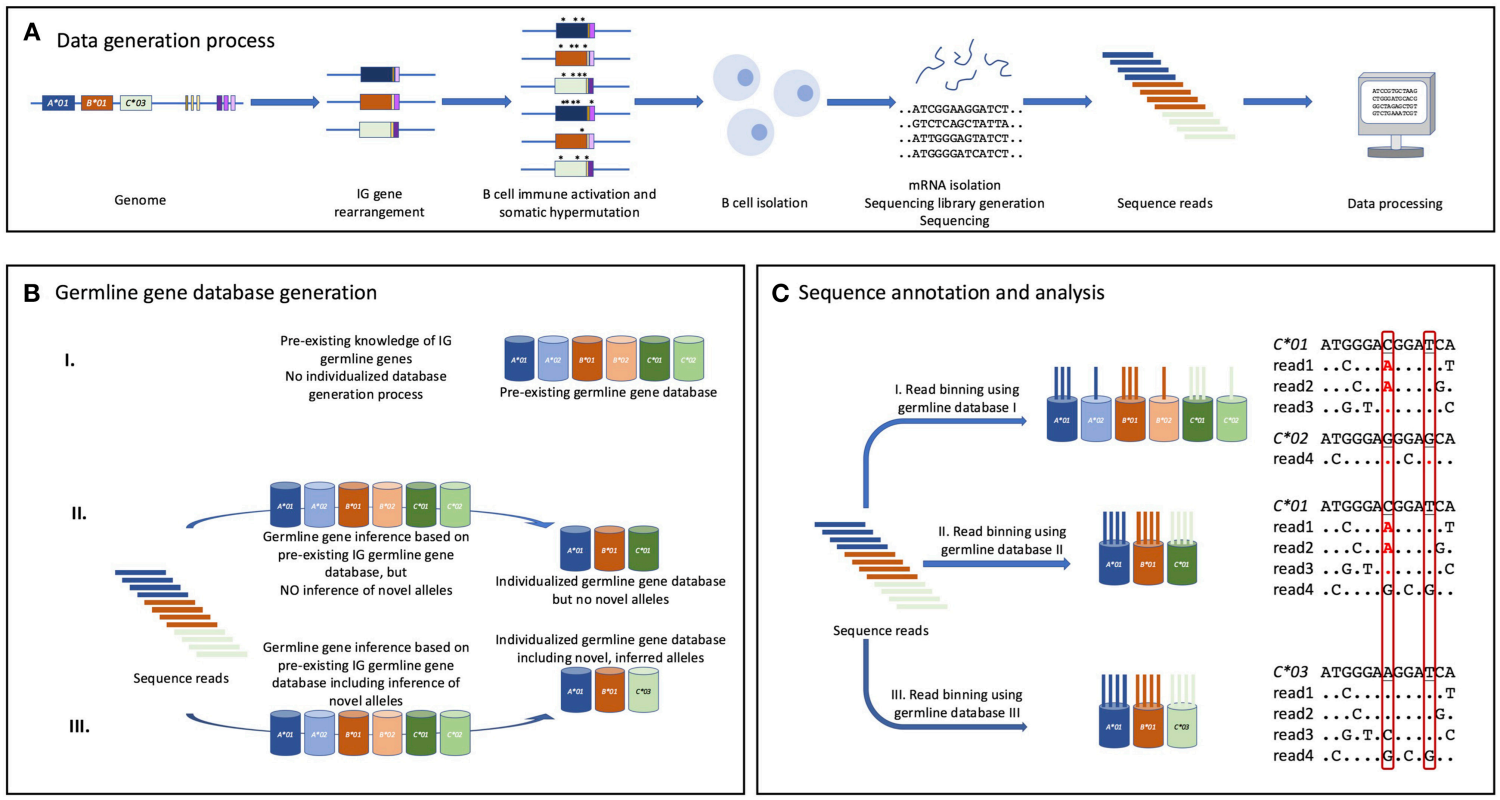
The value of germline IGHV gene inference for detailed AIRR-seq annotation and analysis. **(A)** Germline genes of an individual [here represented by a very limited set of three IGHV genes (A, B, C), and a small number of IGHD (yellow/brown) and IGHJ (purple) genes] are rearranged in cells of the B cell lineage. Following stimulation with antigen many sequences undergo somatic hypermutation and acquire base substitutions (marked ^*^) that may impact subsequent data analysis. An investigated subject's B and plasma cells are collected and typically the cells' transcriptomes are sequenced (e.g., using Illumina MiSeq technology) to generate reads that can be computationally processed. **(B)** A germline IGHV gene database [here represented only by three genes (A, B, C)] will facilitate data analysis, though it is possible to infer genes and alleles without reference to a starting database. The database could be a collection of all known germline IGHV gene alleles (I), or an individualized subset of these (II) that best fits the set of sequence reads that are to be analyzed. Finally, computationally inferred novel germline IGHV gene alleles can be introduced into the individualized germline gene database (III) to even better account for the diversity observed in the experimentally generated sequence dataset. **(C)** Each sequence read is binned to the most appropriate germline gene/allele available in the used germline gene database. If germline gene alleles are present in the database but not in the subject's genotype, some reads will be binned to them as a consequence of base changes introduced by somatic hypermutation (or sequencing errors), resulting in a partial incorrect assignment of germline gene allele origin and consequently of the associated analysis of the mutational pattern. Detailed annotations of part of the sequences are provided for reads binned to alleles of gene *C*. In this example, the investigated subject has an allele (C^*^03) of this gene that is not represented in the original pre-existing germline IGHV gene database. Two bases that differ between one or several alleles in the database and C^*^03, and thus may be misinterpreted in mutational analysis, are boxed. Unless valid inference of novel germline genes is also performed, the mutational analysis will substantially misinterpret the mutational pattern (highlighted in red letters/dots) targeting this gene. Dots indicate identity to the germline gene to which it is compared.

Immunoglobulins (IG) are the main antigen receptors and effector molecules of the B cell lineage, and are expressed either as a component of the membrane-bound B cell receptor (BCR) or as secreted antibodies. They are encoded by large numbers of variable (V), diversity (D), and joining (J) genes, which recombine in developing B cells to generate rearranged V-(D)-J genes. This process, referred to as V-(D)-J rearrangement, occurs at the DNA level and leads to an IG V domain repertoire of immense diversity. The study of such repertoires has recently been revolutionized by high-throughput sequencing (1–4), and this is termed Adaptive Immune Receptor Repertoire (AIRR) sequencing (AIRR-seq). The technical and biological interpretation of AIRR-seq data is facilitated by databases containing reference sequences of all known germline genes (Figure 1), but AIRR-seq studies have demonstrated that these databases are presently far from complete (5–8). This compromises analysis of AIRR-seq data in many ways. For example, it can lead to the inaccurate determination of gene utilization frequencies, and the extent to which sequences have been affected by the process of somatic point mutation.

The first complete nucleotide sequence of a human germline heavy chain variable gene was reported in 1980 (9). In 1989 at the Human Gene Mapping (HGM) (10) Workshop in New Haven, starting with the human T cell receptor gamma (TRG) locus genes as a paradigm, the variable, diversity and joining IG and TR genes were officially acknowledged as “genes” just like conventional genes, and under the HGM auspices, IMGT^®^, the international ImMunoGeneTics information system^®^ (IMGT) was created by University of Montpellier and the Centre National de la Recherche Scientifique (CNRS) (10). Ten years of IMGT biocuration on sequences from human genomic cosmid and artificial chromosome libraries were key to the assembly of the IG loci and their annotation (11–13). The IG and TR gene names, available on the IMGT web site since 1995, were approved by the HUGO Nomenclature Committee (HGNC) in 1999 and are managed by the IMGT Nomenclature Committee (IMGT-NC), the IG, TR and MH nomenclature subcommittee of the International Union of Immunological Societies (IUIS). The functional and open reading frame (ORF) of approved human genes were published with their alleles (203 IG and 168 TR) in two FactsBooks in 2001 (14, 15), and the number of sequences now cataloged by IMGT is shown in Table 1.

**Table 1:** Numbers of human IGHV genes and alleles reported in the IMGT repertoire and in the IgPdb database of inferred alleles.

	**IMGT^a^**		**IgPdb^b^**	
**Subgroup**	**Genes**	**Alleles**	**Genes**	**Alleles**
IGHV1	12	45	8	21
IGHV2	4	29	2	4
IGHV3	30	110	11	18
IGHV4	11	79	8	13
IGHV5	2	9	1	2
IGHV6	1	2	0	0
IGHV7	2	6	0	0

a *IMGT genes and allele counts include sequences reported as Functional sequences and Open Reading Frames. The IMGT repertoire was accessed on 11/02/2019*.

b Sequences in IgPdb that have only been identified by genomic sequencing, and sequences that extend previously reported but truncated sequences are not included. Eleven sequences (IGHV1-2^*^05, IGHV1-2^*^06, IGHV1-8^*^03, IGHV1-69^*^15, IGHV1-69^*^17, IGHV2-70^*^15, IGHV3-11^*^05, IGHV3-11^*^06, IGHV3-13^*^05, IGHV3-43D^*^04 and IGHV3-64D^*^06) that were first discovered by inference but are now present in the IMGT repertoire are also not included here.

With this description of the human IG germline genes, the gene identification and mutation description became an integral part of the study of V-(D)-J gene rearrangements. Over the next 20 years, hundreds of thousands of expressed V-(D)-J genes were reported, and dedicated tools and databases were established to facilitate research (10, 16, 17). It soon became possible to compile datasets of hundreds of rearranged human V-(D)-J gene sequences that could be used to analyse the process of V-(D)-J recombination (18, 19). These analyses also demonstrated that such datasets could be used to identify previously unreported allelic variants of known germline IG genes (20).

**Figure 2:**
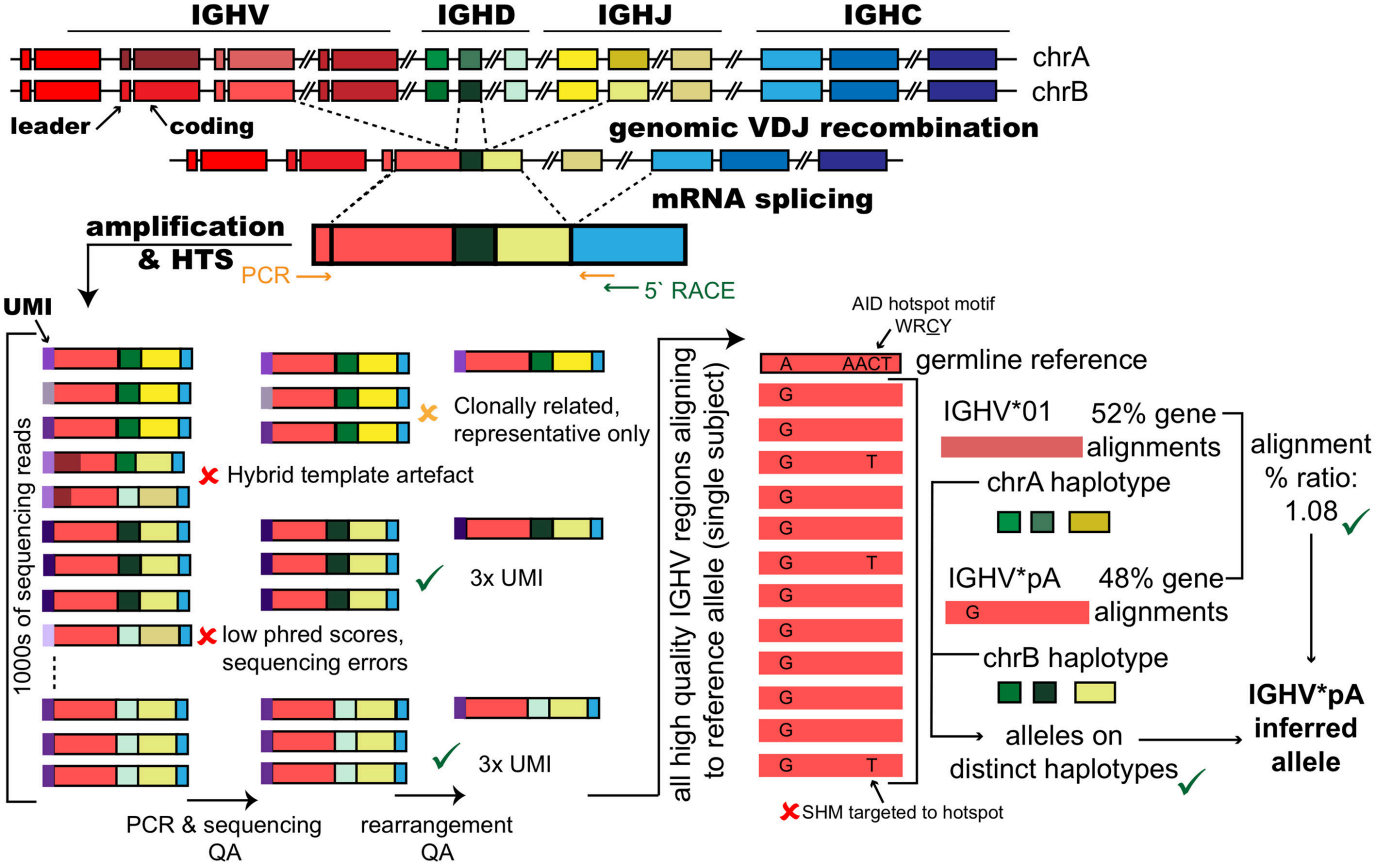
Inference of polymorphic IGHV gene alleles from immunoglobulin repertoire sequencing datasets. The genes that encode immunoglobulin heavy chain variable regions are generated through genomic recombination of single genes of three different types; variable (IGHV), diversity (IGHD), and joining (IGHJ). Each of these gene types are present in the genome as a set of tandem genes that are both polymorphic and polygenic and include approximately 50 IGHV genes, 27 IGHD genes and 6 IGHJ genes. At the mRNA level, splicing joins the rearranged V-(D)-J gene with the IGH constant region genes that confer the isotype to the IG. Repertoire sequencing studies amplify mRNA transcripts, via cDNA, by methods such as targeted PCR using leader region forward primers coupled with reverse primers specific for the IGHC or by using 5′ RACE primed from the IGHC genes. To be suitable for inference, amplification protocols must capture the complete V-(D)-J rearranged coding region and cannot use primer that bind within the V region. Amplification strategies may add unique molecular identifiers (UMIs) as part of cDNA synthesis to tag individual RNA transcripts. High throughput sequencing of a V-(D)-J gene library can generate many thousands of reads for single subjects. Reads are quality assured (QA) to remove sequencing errors; for example, reads with low quality (phred) scores, and PCR artifacts such as hybrid amplicons that have resulted from single stranded DNA from previous PCR cycles acting as primers in future cycles creating hybrid or chimeric templates that are derived from two original amplicons. For inference, IGH from clonally expanded B cells, which have each originated from a single progenitor cell must be reduced to a single representative sequence (for example, the clone member with the fewest mutations) to prevent over-counting. IGH carrying the same UMI can greatly increase confidence that the V-(D)-J rearrangement was in the original pool. In the absence of UMIs, read counts for unique IGH can provide some confidence. Finally, V-(D)-Js are aligned to germline reference datasets that report alleles for the population. Shared “mismatches” relative to the closest germline gene among many sequences from the same subject at position(s) that are not motifs for somatic hypermutation (SHM) in sequences expected to be unmutated (naïve B cells) or have low mutation (IgM+) can be suggestive of the putative allelic variant in the subject's genotype. When a polymorphism is inferred at a heterozygous locus, confidence in the inference can be greatly increased if the putative allele is considered in the context of any other expressed alleles for the gene, with approximately equal expression of the two alleles, and the haplotype, which can phase the gene alleles to their respective chromosomes.

In 2009, AIRR-seq data were reported for the first time (21, 22). Even in the earliest AIRR-seq studies, thousands of independent V-(D)-J rearrangements could be identified from each subject investigated, and this facilitated the detection of previously unreported polymorphisms (5–8) (Figure 2). New allelic variants of IGHV genes were detectable in these AIRR-seq data because the crucial nucleotides that defined these alleles showed up as conspicuous patterns of shared mismatches within alignments to the known germline V gene sequences.

Utilities have now been developed to streamline the identification of allelic variants, and to assign measures of confidence to each inference (23–27). These utilities employ a variety of inference methodologies, as they have been designed for the analysis of different kinds of data. IgDiscover, for example, is best suited to the analysis of relatively unmutated sequences (23), whereas TIgGER (24) and partis (26) are specifically designed to analyse data that include both unmutated and mutated sequences. To date, 58 sequences have been inferred in this way (see Table 1), and can be found in the Immunoglobulin Polymorphism database (IgPdb) (http://cgi.cse.unsw.edu.au/~ihmmune/IgPdb/).

The identification of these previously unreported polymorphisms has remained unknown to many researchers because such variants lie outside the scope of the widely-used IMGT/V-QUEST reference directory of germline sequences (28). This emerged as an early concern of the AIRR Community (https://www.antibodysociety.org/the-airr-community/), a grassroots organization that was founded in 2015 to address the challenges surrounding the generation, analysis and use of AIRR-seq data (29). In 2018, this community formally joined The Antibody Society, a non-profit trade association dedicated to the field of antibody research and immunotherapeutics.

In 2017, the AIRR Community and IMGT agreed to an approach for evaluating the veracity of inferred germline-gene sequences, and for the incorporation of validated sequences into the IMGT Reference Directory. The Germline Database (GLDB) Working Group of the AIRR Community was formed to develop the necessary policies and procedures, and the Inferred Allele Review Committee (IARC) was formed to critically evaluate submitted inferences.

Here we present challenges faced in inferring novel IGHV sequences from AIRR-seq data, and outline strategies for their mitigation. The process for submitting inferred sequences to the IARC is also described. It is our aim that this initiative of the AIRR Community will contribute to a more complete description of human genetic variation, thereby improving the quality of AIRR-seq studies. Human IGHV genes are the focus of this discussion, though the challenges surrounding the inference of other IG and TR germline genes in human and non-human species are likely to be similar. We anticipate that over time this initiative will expand to the documentation of IG and TR genes in all vertebrate species.

## Germline Gene Inferences: Challenges, and Strategies for Minimizing Erroneous Inferences

Reports of inferred antibody sequences have not been immediately and universally accepted, in part because alternative explanations can account for observed nucleotide differences in IG genes (see Figure 2). Uniquely, IG genes within activated B cells undergo secondary diversification by somatic hypermutation (SHM) (30). During an immune response, an IGHV gene with a 300 bp length will commonly accumulate 15–20 somatic point mutations (31, 32) and much higher levels of mutations can be observed (33).

The datasets of Sanger sequences that underpinned the first inferred IGHV sequences were very small—in some cases, just six or seven sequences (20). This raised the possibility that these sequences were mutated versions of known alleles. Importantly though, many of the early inferences have now been confirmed by genomic sequencing (20, 34, 35), lending support to the validity of the inference process. Today, the availability of large AIRR-seq datasets gives much greater confidence in the inference process, but challenges remain. These challenges have their origins in the biology of the B cell and of the antibody repertoire, as well as in technical issues affecting the preparation and sequencing of recombined V-(D)-J gene libraries.

The following strategies and tests will aid in the identification of real allelic variants while minimizing the reporting of erroneous inferences.

**Figure 3:**
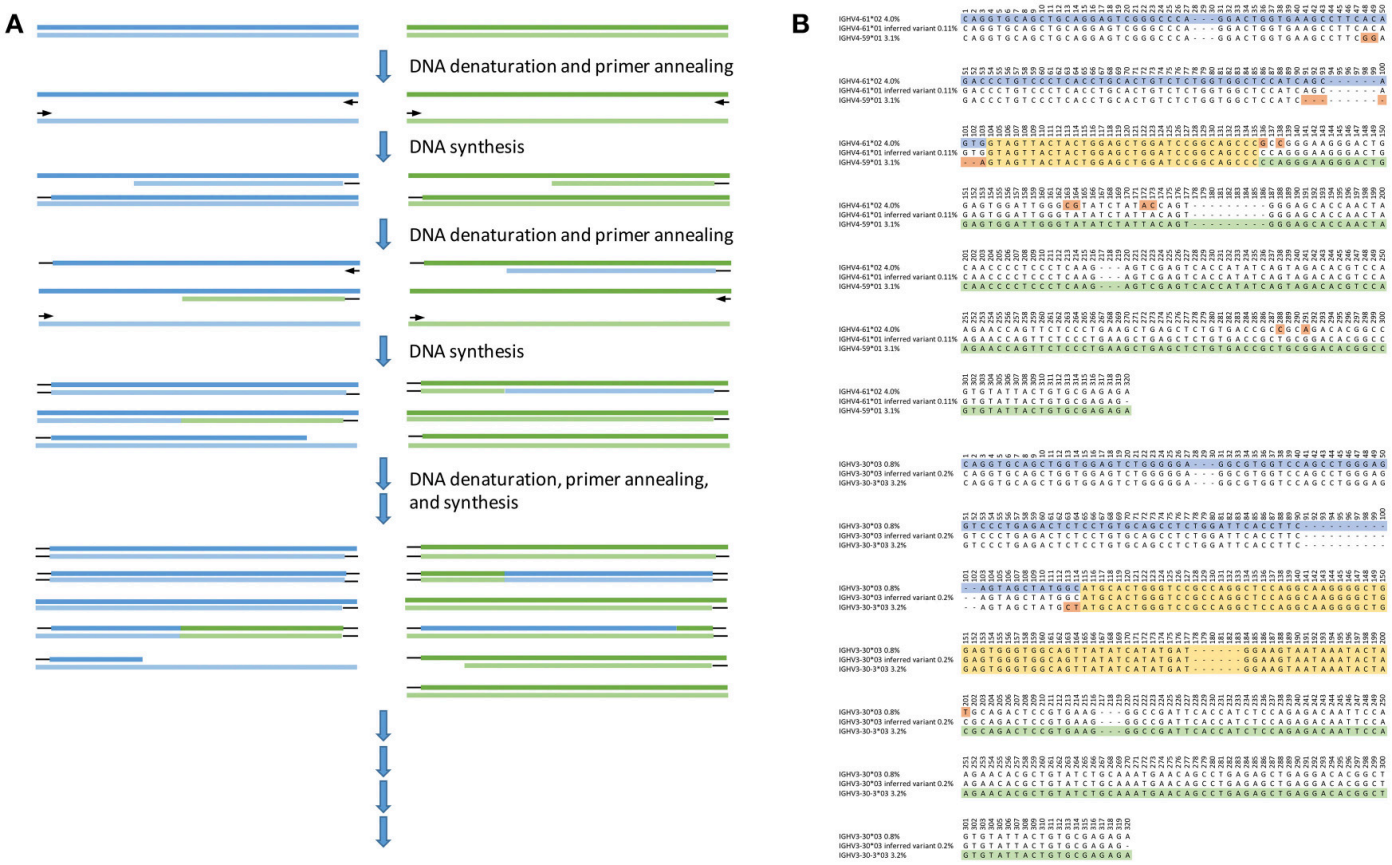
Likely principle of generation of hybrid molecules through cross-over caused by incomplete products during previous PCR cycles **(A)**, and examples of inferred sequences **(B)** that may have an origin in cross-over events during the library generation process. Shown are the inferred sequences (middle) and the parts of other germline sequences that are inferred to be present in the same subject and that through an artifactual cross-over event may have created the inferred novel allele. The region in which cross-over events may have occurred is shown in yellow. The 5′- and 3′- sequences unique to one of the potential donor sequences are shown in blue and green, respectively. Mismatches between the two potential donor sequences are highlighted in orange. The frequency of reads associated with each inference is shown after the name, indicating that some but not all of these inferences may be removed by cut-offs defined in terms of allelic ratios. Base numbering is according to the IMGT numbering scheme.

- Inferences must be made from AIRR-seq data of the highest quality. Experimental strategies to ensure such quality in library generation and sequencing of IG transcripts are now well-established (36, 37), and the assessment of the quality of library generation and of sequencing, using synthetic mRNA spike-ins, is a strategy that can build confidence in inferences made from a dataset (38–41). Proof-reading enzymes with minimal error rates should always be used (42), and putative polymorphisms should be assessed in light of the different types of sequencing errors (base insertions, deletions and substitutions) that are associated with the different sequencing technologies (43). Such errors can be specifically enriched at particular sequence motifs (44), and if these motifs are present in a germline gene, the errors may suggest the existence of a novel allele (7, 45).
- A vital step in the pre-processing of raw sequence data is the removal of reads with a low average quality, but Phred scores should also be assessed for critical nucleotides in individual reads that have contributed to a particular inference. Poor read quality of single nucleotides may result in erroneous inferences (7, 45).
- Correction of sequencing errors and PCR artifacts can be achieved by the use of unique molecular identifiers (UMI). UMIs are introduced during library preparation, labeling each individual transcript prior to amplification. Subsequent consensus building of reads employing identical UMIs can largely remove erroneous bases (46). Technical or biological replicates can also be used to validate sequences and increase confidence that artifacts have been properly discarded.
- Incomplete PCR amplifications create problems. An incompletely amplified product generated in one cycle may later anneal to a similar but distinct template, resulting in the amplification of a hybrid sequence (Figure 3) (47, 48). Such chimeric amplification products are often observed in datasets of IG transcripts (49), and unless appropriate filters are applied to AIRR-seq data, these chimeras can masquerade as novel alleles. Preparing libraries with minimally detectable PCR bands helps reduce the problem of chimerism (49, 50), but this strategy is incompatible with some research objectives.
- The detection and elimination of chimeric sequences can be a valuable step in the pre-processing of data. Manual identification of chimeric sequences involves assessment of the distribution of apparent mutations along the length of a sequence. Chimeric sequences often appear to have somatic point mutations clustered at one or the other end of the sequence, and utilities have been developed to automate the detection of sequences with such a non-random distribution of apparent mutations (51).
- Very large AIRR-seq datasets are required if variants of some IGHV genes are to be identified. Reports from analysis of peripheral blood B cells show that usage frequencies of particular IGHV genes in V-(D)-J rearrangements can be as high as 20% for IGHV3-23^*^01 (52), but as low as 0.01% for rearranged genes incorporating IGHV3-13, IGHV4-28, or IGHV7-81 (5). Rarely utilized IGHV genes will only be present in convincing numbers in the very largest V-(D)-J datasets. Large datasets are also needed if the final nucleotides of a germline IGHV sequence are to be determined. The uncertainties surrounding the nucleotides at the 3′ end of the sequence are a consequence of the variability of the gene ends, produced by the processes of exonuclease removal and N nucleotide addition. Biases in these processes can result in the generation of relatively common motifs that may be mistaken for germline-encoded nucleotides (53–56). Of special note, the last base of a germline sequence may not be the most common base in rearranged sequences.
- Somatic point mutations accumulate in IG-encoding genes at a rate of about one mutation per 1,000 bp per cell division within the germinal center reaction (57, 58). The existence of mutational hotspots (59–61) that can target specific germline IGHV genes (62, 63) means that it is inevitable that there will be some shared mutations in any dataset that includes mutated sequences. Some IGHV genes have positions that can be mutated in >30% of class-switched sequences (24). Very high levels of mutation can occur at positions far removed from the regions encoding complementarity determining regions (CDR) of an IGHV sequence, and even at positions outside conventional mutational hotspots (62, 64). For these reasons, inferences of new germline IGHV genes using datasets of mutated sequences are more likely to be erroneous.

Somatic point mutations may be mistaken for germline-encoded nucleotides, but this issue is substantially reduced if sequences are derived from less-mutated cell populations. This can be achieved by the amplification of IgM-encoding transcripts through the use of constant region-specific primers. The issue is partially addressed by the amplification of sequences from sorted B cells displaying a naïve phenotype. More highly mutated datasets can, however, still be the source of reliable inferences if appropriate analytical tools are used. Both the TIgGER and partis software suites, for instance, are designed to use patterns of apparent mutation to infer novel alleles (24, 26). While taking different overall approaches, they both use regression-based statistical tests to identify polymorphisms at positions that appear to be recurrently mutated, in sequences that are otherwise relatively unmutated.

**Figure 4:**
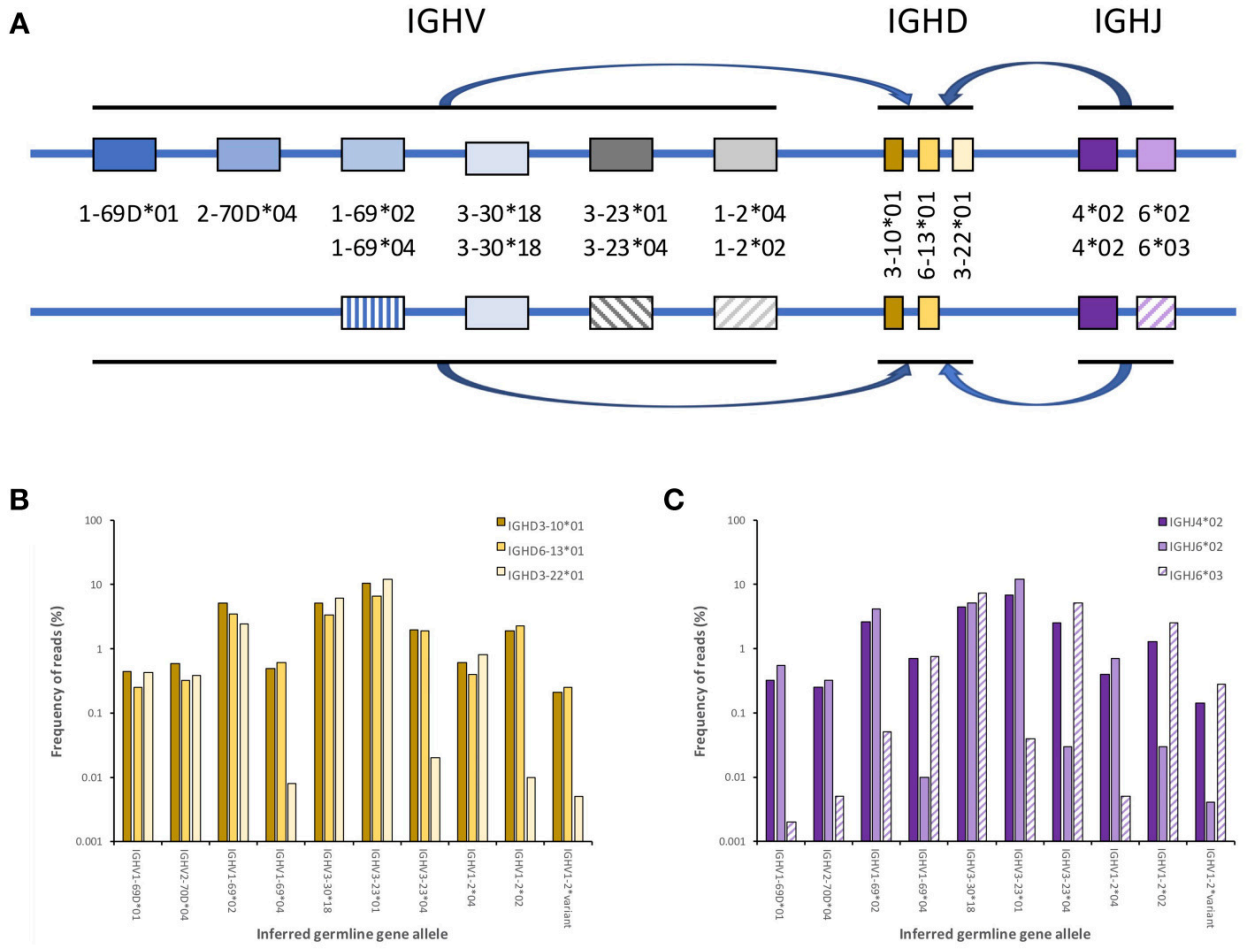
Principle of inference haplotyping defining the AIRR-encoding genes associated with each of the chromosomes carrying the relevant locus, here demonstrated by genes encoding human IG heavy chains. Germline genes involved in V-(D)-J rearrangements are from genes harbored on each chromosome only (i.e., in *cis*), as illustrated with a small number of the genes that actually populate the IGH locus **(A)**. It is possible, using large sequence datasets, to computationally define the association of each IGHV allele to one of the two haplotypes using their association to e.g., different alleles of an IGHJ gene (commonly IGHJ6), if such different alleles are present in the genotype. These alleles serve as anchors in the haplotyping process. An IGHV allele that resides on both chromosomes will rearrange to both alleles of the heterozygous IGHJ gene, whereas IGHV alleles that reside on only one chromosome should primarily be found rearranged to one of the alleles of the heterozygous IGHJ gene **(C)**. In the case of haplotype differentiating expression of IGHD genes (or allelic differences in one or several IGHD genes) these differences can similarly be used as anchors to visualize IGHV allele distributions between haplotypes **(B)**. Such inference can be used to raise confidence in specific allele calls, as incorrectly inferred alleles are likely to associate with the same haplotype as another allele of the same (or a very similar allele) that also exist in the haplotype. This is exemplified here by the haplotyping of an artifactual inference of a novel allele (IGHV1-2^*^variant) that has a similar association to haplotypes as IGHV1-2^*^02. [For specific examples, see Kirik et al. (22, 53)].

- A useful test of an inferred allelic variant is to consider the sequence in the context of other alleles of the gene that may be present in the dataset. For single-copy genes, only two alleles should be present in the genotype of an individual. If an inference suggests that three alleles of a particular gene are present in an individual genotype, the inference should be further investigated. More than two named variants of some genes can be present in an AIRR-seq dataset for gene names without genomic information on the haplotype copy number, as a result of copy number variation (CNV) (5, 6, 8, 35, 46, 65). It is highly likely, for example, that some named allelic variants of the IGHV1-69 gene are actually variants of the duplicate IGHV1-69D gene. Genotypes may also include three or more alleles of one or other of the highly similar IGHV4-4, IGHV4-59, and IGHV4-61 genes, for the genomic location of some sequences associated with these genes is uncertain (7, 45, 52, 66). It seems likely, for example, that the IGHV4-59^*^08 sequence in some subjects is actually a variant of the IGHV4-61 gene (66). In view of these complications, an evaluation of some inferences must be made with reference to alleles of several genes that may be present in the genotype of the individual. Genomic data of a single cell or individual will remain necessary to unambiguously assign expressed genes with CNV.
- Alleles at heterozygous loci are usually expressed at similar frequencies (52), while inferred sequences suggested by sequencing errors or somatic point mutations are usually present at relatively low frequencies. The calculation of the percentage of alignments to a gene that involve the inferred allele is therefore a simple test that can be used to identify false inferences. Although the IARC will affirm inferred alleles that are observed in just 10% of all alignments to a particular gene, additional analysis may be required to support inferences that imply expression at a low level. We recognize that this will make it more difficult to infer some alleles. CNV, mentioned above, will also complicate the interpretation of this measure of allele expression, whereas recombination signal (RS) sequence variation and other non-coding region variation could lead to abnormal allele expression levels (67). For all these reasons, the allele expression test has limitations.
- The validity of an inference can be demonstrated if all V-(D)-J sequences containing the inference in an AIRR-seq dataset are associated with just one of the two chromosomes of the individual. Such validation can be done using haplotype analysis as outlined in Figure 4. This is a method that was developed for human AIRR-seq studies (6, 53, 68), and it is increasingly being used to support reported inferences (7, 45, 52). Haplotyping is only possible for the validation of IGHV gene inferences in subjects who are heterozygous at IGH loci beyond the IGHV locus region. Anchors for the haplotype inference of IGHV genes are most commonly IGHJ6 alleles (IGHJ6^*^02 and IGHJ6^*^03), but heterozygosity at the IGHD2-8 and IGHD2-21 loci can also allow them to be used (7, 45, 52, 68). It is likely that novel long-read high-throughput sequencing platforms will soon make it possible to use IGH constant region genes as haplotype anchors as well.

## Submission of Inferences and Data Deposition

IARC and the GLDB WG strive to provide the community with open, transparent and reusable information on inferred genes. To this end, a web-based service termed Open Germline Reference Database (OGRDB) has been set up to facilitate the submission and evaluation of inferences as well as the subsequent retrieval of inferred genes accepted by IARC. In addition, the inferred sequence and the NGS data supporting it have to be deposited in general purpose sequence repositories of the International Nucleotide Sequence Database Collaboration framework to allow re-analysis by third parties and ensure long-term availability of the data. The detailed workflow for data submission is available at OGRDB (https://ogrdb.airr-community.org). In brief, it covers the following steps:

- Verification that the complete raw data of the underlying experiment is available via the Sequence Read Archive (SRA). If possible, the SRA and associated metadata records should be compliant with the Minimal Information about Adaptive Immune Receptor Repertoire (MiAIRR) standard (69).
- Deposition of reads supporting the gene inference to SRA. Note that this submission is in addition to the publication of the complete read data of a given set of experiments.
- Submission of the inferred sequence to GenBank/TPA, depending on the origin of the data on which the inference is based:
  - First-party data (the inference is performed on one's own datasets) is submitted to GenBank.
  - Third-party data (inference performed on datasets produced by others) is submitted to GenBank's Third Party Annotation (TPA) section.
- Submission of the inferred sequence and the associated information about the inference procedure as well as the accession IDs of the INSDC submission to IARC via the OGRDB interface.

Each inference must be made from data that originates from a single individual. The standardized submission protocol incorporates metadata related to the individual, as well as to the generation, processing and analysis of the individual's sequences. It also provides data that gives the genotypic context in which an inference should be assessed, and helps identify confounding factors that should be considered.

Currently, data used for germline IGHV gene inference are often generated from PCR-amplified IG transcripts using Illumina's MiSeq technology, as it provides sufficient read length and depth. The IARC will, however, consider inferences and determinations made in other ways. The IMGT-NC requires genomic sequencing of IGHV genes, including the complete leader sequence and associated Recombination Signal sequence (V-RS). Genomic sequences that are not suitable for submission to IMGT-NC will be considered by the IARC if they include the complete IGHV coding region. Partial genomic sequences may also be considered by IARC as evidence in support of an inference from AIRR-seq data. Direct RNA sequencing (70) may also come to play an important role in defining germline IGHV genes in the future.

Inferences must be made from full-length sequencing reads. In contrast, many studies employ primers that anneal within the IGHV sequences themselves, such as the well-validated BIOMED-2 primer set (71). Although sequences generated in this way may be suitable for many research purposes, the partial sequences that can be inferred from such datasets are not suitable for submission to IARC. Submitted sequences must be full-length V-REGION sequences, from base 1 to at least base 318 of the IGHV sequence, according to the IMGT numbering system. Inferences generated using primers that anneal within the sequence should not be submitted to the IARC.

Inference may be carried out using a diversity of computational methodologies. The IARC is agnostic to the investigator's choice of inference methodology as long as it is validated, published, publicly available, and well-documented.

We believe that the identification of dependable, curated gene sets, to which this effort contributes, is a public good. To that end, affirmed sequences, and the submissions that support them are published by IARC under the Creative Commons CC0 license (https://creativecommons.org/publicdomain/zero/1.0/legalcode), allowing their use for any purpose without restriction under copyright or database law.

## The Evaluation and Decision-Making Process

The affirmation of submitted inferences requires the unanimous support of the IARC, and this may only be possible after the provision of additional information by the Submitter. The deliberations of this Committee may differ depending on the biological context in which particular sequences are observed and on the process of inference. Particular attention will be paid to:

- The frequency of V-(D)-J rearrangements that include the inferred sequence. Inferences that appear to be very rarely represented in the IG repertoire are at high risk of being incorrect inferences. To guard against this, inferences of sequences that are seen at a frequency of 0.05% or less will not generally be affirmed.
- The number and frequency of unmutated sequences representing the inferred sequence.
- The presence of the inferred IGHV sequence in a diversity of V-(D)-J rearrangements. The sequence needs to be seen in association with different IGHJ genes and in rearrangements with varying CDR3 lengths. This guards against the possibility that sequences that support the inference are clonally-related sequences.
- The number of alleles assigned to the relevant gene or to the set of highly similar genes.
- The distribution of reads between an inferred allele and other alleles of that particular gene, calculated using unmutated sequences. Inferences with low expression frequencies may require additional supporting evidence.
- The outcome of haplotype analysis, where such analysis is possible.
- Evidence that PCR artifacts, such as cross-over events involving other genes and alleles of the subject's genotype, do not explain the inference. Evidence could include a demonstration of the absence of cross-over effects in sequencing libraries of germline gene standards analyzed in parallel to the subject's expressed IG repertoire (38), or demonstration of the systematic identification and removal of sequences with evidence of cross-over effects prior to inference, or analysis of the extent of shared CDR3 sequences between different V-(D)-J gene rearrangements.
- Evidence supporting the reported 3′-end of an inferred germline IGHV gene. The final base of an IGHV gene sequence cannot be inferred with confidence (55, 56) unless additional investigations are undertaken. If a sequence is reported up to and including base 320, the final base will only be affirmed by IARC if supporting analysis is provided.
- Sequencing of part of an inferred allele, from non-B cell genomic DNA.

An assessment will result in one of three outcomes. If a sequence is affirmed as a valid inference, it will be assigned an IARC sequence name and a summary of evidence in support of the inference will be documented in an Inferred Sequence Documentation Sheet. This will be made publicly available at the AIRR community website. It will also be reported to IMGT-NC with an individual GenBank accession number for inclusion in the IMGT Reference Directory. When a sequence is affirmed for the first time, it will be reported as a Level 1 Sequence. If affirmed a second time, it will be reported as a Level 2 Sequence, and if affirmed a third time, it will be reported as a Level 3 Sequence. It is important that researchers continue to notify the IARC of later identification of Level 1 and Level 2 Sequences, so that they can rise to higher tiers. This will promote acceptance of the inferences within the research community. The IARC will not consider additional inferences of a sequence following its elevation to Level 3.

If evidence in support of a sequence does not reach the level of certainty required for immediate affirmation, the sequence may remain “under review”. An Inferred Sequence Documentation Sheet will be completed, and the sequence will be assigned an IARC name, but it will not be publicly reported. Such sequences will be re-assessed if additional supporting information becomes available, or if identical inferences are later submitted to IARC. If a later inference supports the elevation of the sequence to Level 1, the original inference will be credited in the documentation of the sequence.

If there is insufficient evidence to allow a sequence to remain “under review”, details of the submission will be retained by IARC, but the submission will not be a part of any future re-assessments.

Inferred alleles will be named using a modification of the IMGT nomenclature (72), incorporating:

- the gene locus (e.g., IGHV, IGKV, IGLV for genes of heavy, kappa light, and lambda light chain loci, respectively);
- the most similar gene at the time of submission in the IMGT/V-QUEST reference directory (28), or in the case of multiple, most-similar genes, using the name with the lowest alphanumeric value;
- an allele number, preceded by an “i” to indicate its discovery by inference. Assigned allele numbers for any gene will be consecutive, and the first inferred allele will be designated the ^*^i01 allele (e.g., IGHV1-2^*^i01).

A given allele number for a specific gene will be uniquely associated with a specific sequence. If the sequence is incorporated into the IMGT Reference Directory, it will be assigned a new name by IMGT-NC based on the chronological rule and reported to the IUIS/IMGT Nomenclature Committee. The inferred allele name will not be reused and records of the inference will be permanently maintained. Similarly, if evidence emerges suggesting that a particular inference was made in error, the sequence will be removed from any listing of affirmed sequences, but the name and documentation sheets will remain permanently associated with the sequence.

**Figure 5:**
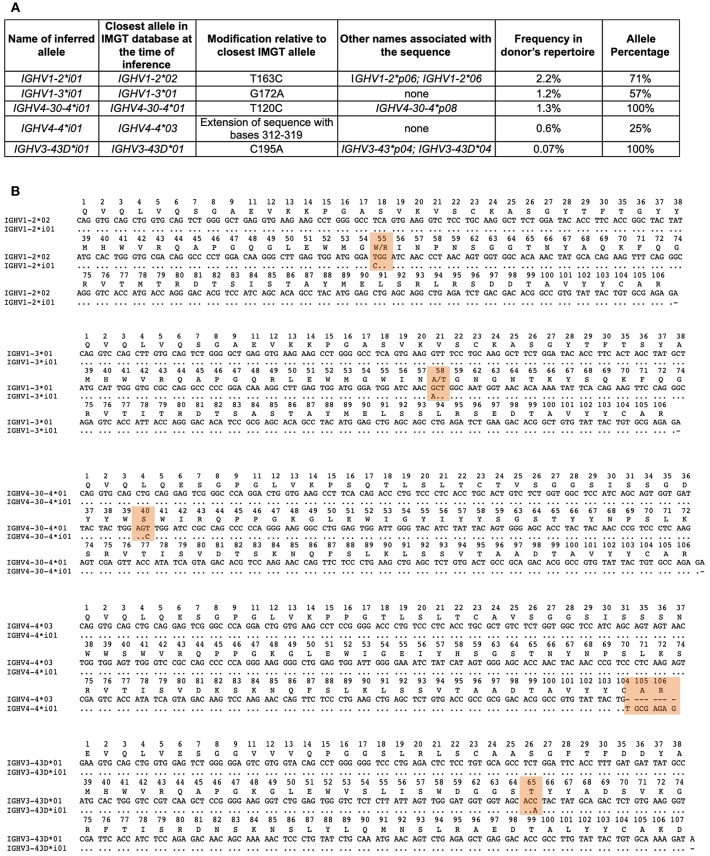
Affirmed inferred alleles. **(A)** Table of inferred alleles. Shown are the names given to the inferred sequences by IARC; the closest matching IMGT alleles; the genetic differences observed in the inferred allele relative to the IMGT allele; any other name that has previously been associated with this sequence, if previously identified; the genotype frequency of the inferred sequences within the donor's genotype and the allele percentage of the inferred allele based on all of the alleles within the donors genotype for that particular gene. **(B)** Alignment of each inferred sequence relative to the closest matching IMGT allele with the differences between the sequences highlighted in orange. Numbering of the alignments are according to IMGT numbering.

Germline gene databases currently include entries that are incomplete at the 5′ and/or the 3′ end. The inference process could allow the extension of incomplete sequences, as is the case with the sequence IGHV4-4^*^i01 that is reported here (see Figure 5). A sequence of this kind could be a longer representation of the previously reported allele, or it could be a very similar sequence that varies from the original sequence at its ends. The IARC will not attempt to resolve this ambiguity and will simply assign an inferred allele name to the new sequence.

## Affirmed Novel Alleles

Using the recommendations and policies outlined above, as of August 31, 2018, the IARC has approved five novel alleles at Level 1 (Figure 5) and nine inferred alleles remain “under review” (data not shown). Four of the inferred alleles were affirmed from data submitted by the data-generating author (73), of which three were from one donor and one was from a second donor.

IGHV1-2^*^i01 differs from IGHV1-2^*^02, its closest matching allele from IMGT, by a single substitution (t163c), resulting in an amino acid change (W55R). Exact matches to the inference were seen in 2.19% of those donor sequences that were determined to be unmutated rearrangements. A second allele for IGHV1-2 (IGHV1-2^*^04) was observed within the subject's genotype, however IGHV1-2^*^i01 was seen in 71% of alignments to IGHV1-2. This sequence has been previously described in multiple subjects from AIRR-seq (5, 7, 24), and from genomic DNA (8) and it is listed in the IgPdb database as IGHV1-2^*^p06. Since this inference was affirmed by IARC, it has been confirmed using full-length genomic DNA sequencing and was recently accepted (24 July 2018) by IMGT-NC as IGHV1-2^*^06 (Report 2018-1-0724) (http://www.imgt.org/IMGTindex/IMGT-NC.php).

IGHV1-3^*^i01 was present in 1.17% of the donor's sequences, and differs from IGHV1-3^*^01 by a single nucleotide (g172a), resulting in an amino acid change (A58T). This sequence has not been observed previously.

IGHV4-30-4^*^i01 was observed in 1.3% of the donor's sequences, and also has a single nucleotide difference (t120c) compared to its closest matching IMGT allele, IGHV4-30-4^*^01, however this did not result in an amino acid change. It has been observed in multiple individuals from genomic DNA sequencing (8) and in a single individual from AIRR-seq (63). It was previously listed as IGHV4-30-4^*^p08 in the IgPDb database.

IGHV4-4^*^i01 was observed in 0.6% of the donor's sequences. It may be an extension of the existing IGHV4-4^*^03 allele described in IMGT, involving bases 312-319.

The last of the five affirmed alleles, IGHV3-43D^*^i01, was submitted as a third party annotation dataset (74) and although it was observed at a low frequency (0.07%) in the subject's repertoire, it could be accepted as a Level 1 sequence. It has been observed previously in multiple individuals from AIRR-seq studies (7), and as genomic DNA (8), and is listed as IGHV3-43^*^p04 in IgPdb. It has also been observed in a fosmid clone (GenBank: AC242184) that was not annotated in detail. At the time of its acceptance by IARC, this sequence differed from its closest matching IMGT sequence IGHV3-43D^*^01 (now renamed as IGHV3-43D^*^03) by a single nucleotide (c195a), however this does not result in an amino acid change. Since the affirmation by IARC of this novel inferred allele, it has been accepted (October 4, 2018) by IMGT as IGHV3-43D^*^04, based on genomic evidence.

For all five affirmed alleles, the genotype and allele frequencies were within the IARC guidelines. Where possible, haplotype analysis confirmed the validity of the inferences, and cross-over artifacts were ruled out. The Inference Documentation Sheets for these inferred alleles can be found at the OGRDB website (https://ogrdb.airr-community.org).

## Conclusion

Germline IGHV, IGHD, and IGHJ genes constitute the building blocks of IG V domain diversity, and so have a direct bearing on the functional B cell immune response. The formation of IARC, and the establishment of processes for the evaluation of inferred sequences provides an important new avenue for cataloging germline gene variation at the population level. Ultimately, this should provide insights into how germline gene diversity influences functional immunity (75, 76). Here, we describe the prerequisites, procedures and potential outcome of the IARC-based review and evaluation process, and as proof of principle, we report five novel alleles.

The establishment of the IARC review process should help the research community to chart germline IGHV gene variation across human ethnicities and patient groups. This is an achievable goal if studies increasingly infer the germline gene repertoires of each of their study subjects. Such personalized references databases will also improve AIRR-seq studies, through the improved germline gene annotation and confidence in identification of SHMs that will result (Figure 1).

The AIRR Community and the IMGT group have attempted to provide a robust roadmap and conceptual framework for germline gene inference, but the challenge will now be to encourage the incorporation of germline gene inference software into preprocessing and data analytical workflows. This has not yet been widely adopted by the community of researchers who generate and analyze AIRR-seq data. To facilitate this, IARC aims to create detailed step-by-step experimental and bioinformatics tutorials, and will document case studies showing the manifold advantages that lie in this approach. To minimize human intervention and subjectivity, we will also work to further automate the evaluation process of putative germline gene alleles, and to improve the data submission toolchains to INSDC repositories and to IMGT. Finally, in the future, we intend to partner with other researchers, to extend this initiative to the validation of other adaptive immune receptor gene loci. Other IG and TR genes in humans and species of medical importance may be an early focus, but in time we anticipate that the process of inference can be used to extend our knowledge of antigen receptor genes in all vertebrate species.

Putative novel alleles may now be submitted to the IARC-managed web portal for evaluation.

## Data Availability

Publicly available datasets were analyzed in this study. This data can be found here: http://ogrdb.airr-community.org/.

## Author Contributions

The authors are all members of the Germline Database Working Group of the AIRR Community of the Antibody Society. All authors contributed to the development of the policies and procedures described. MO and AC drafted the manuscript, and all authors contributed to the editing of the manuscript.

### Conflict of Interest Statement

The authors declare that the research was conducted in the absence of any commercial or financial relationships that could be construed as a potential conflict of interest.
